# Microbial communities of the house fly *Musca domestica* vary with geographical location and habitat

**DOI:** 10.1186/s40168-019-0748-9

**Published:** 2019-11-08

**Authors:** Rahel Park, Maria C. Dzialo, Stijn Spaepen, Donat Nsabimana, Kim Gielens, Herman Devriese, Sam Crauwels, Raul Y. Tito, Jeroen Raes, Bart Lievens, Kevin J. Verstrepen

**Affiliations:** 1 VIB–KU Leuven Center for Microbiology, Gaston Geenslaan 1, 3001 Leuven, Belgium; 2 CMPG Laboratory of Genetics and Genomics, Department M2S, KU Leuven, Gaston Geenslaan 1, 3001 Leuven, Belgium; 3 Leuven Institute for Beer Research (LIBR), Gaston Geenslaan 1, 3001 Leuven, Belgium; 4 Biology Department, School of Science, College of Science and technology, University of Rwanda, RN1, Butare, Rwanda; 5 Safety, Health & Environment Department, UZ Leuven, Herestraat 49, 3000 Leuven, Belgium; 6 Laboratory for Process Microbial Ecology and Bioinspirational Management (PME&BIM), Department M2S, KU Leuven, Campus De Nayer, Fortsesteenweg 30A, 2860 Sint-Katelijne Waver, Belgium; 7 Bioinformatics and (eco-)systems biology lab, Department of Microbiology and Immunology, Rega institute, KU Leuven, Herestraat 49, 3000 Leuven, Belgium

## Abstract

House flies (*Musca domestica*) are widespread, synanthropic filth flies commonly found on decaying matter, garbage, and feces as well as human food. They have been shown to vector microbes, including clinically relevant pathogens. Previous studies have demonstrated that house flies carry a complex and variable prokaryotic microbiota, but the main drivers underlying this variability and the influence of habitat on the microbiota remain understudied. Moreover, the differences between the external and internal microbiota and the eukaryotic components have not been examined. To obtain a comprehensive view of the fly microbiota and its environmental drivers, we sampled over 400 flies from two geographically distinct countries (Belgium and Rwanda) and three different environments—farms, homes, and hospitals. Both the internal as well as external microbiota of the house flies were studied, using amplicon sequencing targeting both bacteria and fungi. Results show that the house fly’s internal bacterial community is very diverse yet relatively consistent across geographic location and habitat, dominated by genera *Staphylococcus* and *Weissella*. The external bacterial community, however, varies with geographic location and habitat. The fly fungal microbiota carries a distinct signature correlating with the country of sampling, with order Capnodiales and genus *Wallemia* dominating Belgian flies and genus *Cladosporium* dominating Rwandan fly samples. Together, our results reveal an intricate country-specific pattern for fungal communities, a relatively stable internal bacterial microbiota and a variable external bacterial microbiota that depends on geographical location and habitat. These findings suggest that vectoring of a wide spectrum of environmental microbes occurs principally through the external fly body surface, while the internal microbiome is likely more limited by fly physiology.

## Introduction

The common house fly, *Musca domestica* L. (Diptera: Muscidae), is a robust commensal organism, capable of surviving in a broad spectrum of environments. *Musca domestica* lives in close proximity to humans and domesticated animals, commonly found in homes, food markets, farms, and ranches, hovering around decaying matter, garbage, feces, and human food [1]. They prefer warmer (optimal 30 °C) and drier conditions but are able to breed at a reduced rate throughout colder seasons, typically in livestock stables [2–4]. The species is found on every continent, except for Antarctica [5].

During each developmental stage (larvae, pupae, adults), house flies are tightly associated with microorganisms [6]. Recent studies have aimed at establishing the microbiota of *M*. *domestica* with sampling from various environments and even different countries. Proteobacteria, Bacteroidetes, and Firmicutes were found as major phyla of the house fly microbiota [7–9].

Flies can spread microbes through excretion, contact with contaminated legs or mouthparts, and by regurgitation while feeding [10, 11]. They move on average a few hundred meters up to several kilometers in a couple of days, even when food and oviposition sites are plentiful [12–14]. House flies have been observed carrying pathogenic *Escherichia coli* from a dairy farm to a restaurant in a town 3 km away [15]. Moreover, some microbes that are transiently associated to the flies can remain alive for days in the mouthparts and crop of the flies [10]. This highlights the capacity of these filth flies to vector microbes between habitats over large distances. A recent review identified over 100 pathogenic bacteria, fungi, parasites, and even viruses that have been found in or on *M*. *domestica* adults and larvae, some of which are potentially antimicrobial-resistant [11, 16].

Detection and identification of microbes associated with *M*. *domestica* has primarily relied on culture-dependent techniques and may therefore not provide an accurate representation of how commonly human pathogens occur on house flies. Culture-independent methods such as amplicon sequencing or shotgun sequencing allow for a more accurate characterization of microbial communities [17]. Additionally, the fungal component of the microbiota is frequently overlooked, possibly because fungi are often associated with insects feeding on wood or detritus [18]. Although some studies have attempted to make geographical comparisons, limited sampling numbers and inconsistencies across sampling habitats have made it difficult to draw conclusions about the impacts of geography and habitat on the house fly-associated microbiota [7, 9].

In this study, we took an in-depth look at the potential influence of geography and habitat on the internal and external microbial communities of *M*. *domestica*. We sampled over 400 flies from two geographically distinct countries, Belgium and Rwanda, and three types of environments, including cow farms, homes, and hospitals. DNA was extracted from the washing liquid of the external surfaces and internal homogenates of each fly. Extracted DNA was subjected to amplicon sequencing of partial bacterial 16S ribosomal RNA (rRNA) genes and the fungal internal transcribed spacer 2 (ITS2) region. Our analysis demonstrated that the internal bacterial community largely overlaps regardless of location, while the external bacterial and fungal (both internal and external) communities vary considerably with the sampling location, with flies from farms carrying the most distinct set of bacterial taxa regardless of country of origin. Furthermore, we identified microbial signatures of each habitat that shed light on the microbe vectoring potential and patterns of *M*. *domestica*.

## Results

### Large-scale sampling of *M*. *domestica* from distinct geographical locations and habitats

Over 400 flies from Belgium and Rwanda were collected and examined in this study (Additional file 2: Table S1). We aimed to collect 15 males and 15 females from different sampling sites spread over two countries (Rwanda and Belgium), and three different environments (cow farms, homes, and hospitals) (Additional file 1: Figure S1A,B). Hereafter “habitat” will denote each of these environments separately in Rwanda and Belgium, and “site” refers to the sampling location. Each habitat had at least three sampling sites except for Belgian hospitals, which proved to be virtually devoid of flying insects. Additionally, as a reference sample, 30 flies were collected post-emergence from a lab-grown strain (WHOij2) which has been raised in laboratory conditions for approximately 70 years.

The internal and external microbiota of the flies was investigated via high-throughput amplicon sequencing of the V4 region of the bacterial 16S rRNA gene and the fungal ITS2 region. Amplicons were sequenced on an Illumina MiSeq platform. After quality filtering and removal of samples with low read numbers (see “Methods” section for details), an average of 24,776 (± 641 SEM) V4 reads and 24,999 (± 906 SEM) ITS2 reads were retained per fly sample.

### Diversity of the bacterial and fungal communities from *M*. *domestica*

**Fig. 1:**
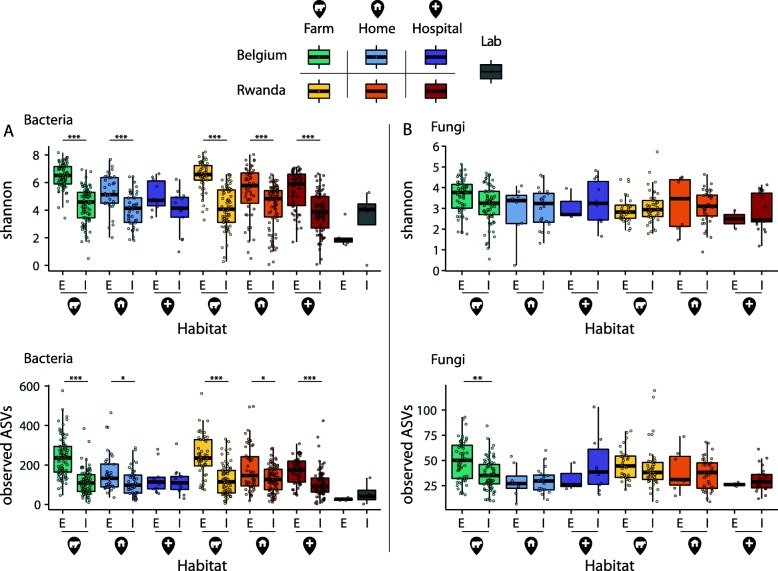
Boxplots showing the alpha diversity comparisons of the external and internal house fly samples. The upper and lower whiskers correspond to the first and third quartiles, with the bar in the middle marking the median value; the dots indicate the value of each data point. Alpha diversity was measured by Shannon index (top panels) and observed amplicon sequence variants (ASVs) (bottom panels) for **a** bacteria and **b** fungi. Each sampling site is colored according to habitat and country as indicated in the key. Significant differences between corresponding external (“E”) and internal (“I”) communities are depicted; *p.*adj* < 0.05, **p.*adj* < 0.01, *** *p*.*adj* < 0.001

Alpha diversity comparisons of the bacterial communities revealed that regardless of country or habitat, house flies harbor a highly diverse bacterial microbiota, with the external bacterial communities being even more diverse than the internal populations (overall median Shannon diversity 6.2 [range 0.5–8.2] versus 4.3 [range 0.004–6.9], respectively) (Fig. 1a Additional file 3: Table S2). It is possible that some of the external diversity could be due to transfer from fecal deposition during the collection process; however, the higher diversity of external samples still indicates that the external surface of flies harbors additional taxa compared to the internal environment. This is supported by the observation that among the most common amplicon sequence variants (ASVs), defined by presence in at least 10% of samples, there are 141 ASVs that are unique to the external surface compared to 26 ASVs unique to the internal compartment (Additional file 1: Figure S2A). There were two notable exceptions to this trend: lab-grown flies and Belgian hospital flies had relatively low alpha diversities and no significant difference between internal and external compartments, which might be linked to the cleaner environment in which these flies live (Fig. 1a).

The overall diversities of the samples from the two countries investigated were relatively similar, with median Shannon diversity of 5.2 in Belgium and 5.0 in Rwanda (Additional file 3: Table S2). Interestingly, less than half of the common bacterial ASVs overlapped between the two countries, suggesting that while the diversities are similar, there are differences in the composition of the bacterial taxa (Additional file 1: Figure S2B).

To assess the effect of sampling environment on alpha diversities, fly samples from farms, homes, and hospitals (combined external and internal samples from both countries) were compared. The farm samples were significantly more diverse than both home and hospital samples, while between homes and hospitals the diversities were relatively similar (Additional file 3: Table S2, Additional file 1: Figure S2C).

Fungal communities showed on average 2.5 times less observed ASVs than the bacterial communities. The median Shannon diversity of fungal communities from the internal compartment was 3.1 (range 0.6–5.7), and 3.3 (range 0.2–5.1) from the external compartment, with less variation between habitats (Fig. 1b). The lab-grown flies had no detectable fungal amplicons.

### Geography and habitat influence the composition of *M*. *domestica* microbial communities

**Fig. 2:**
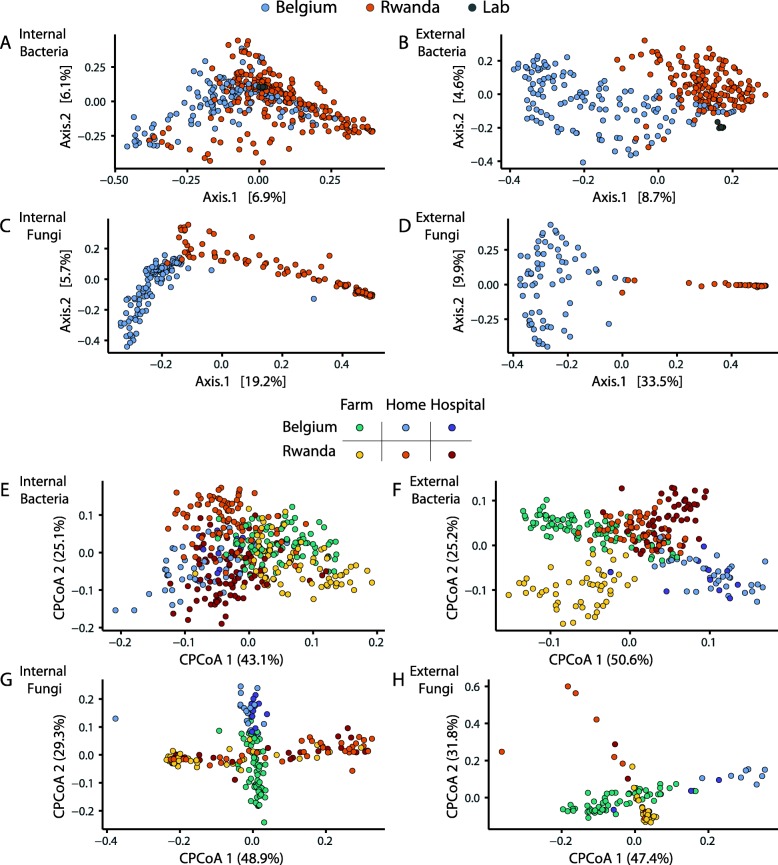
PCoA analysis demonstrating the influence of geography and habitat on bacterial and fungal communities. **a**–**d** Principle coordinates analysis of Bray-Curtis distances of **a** internal bacteria, **b** external bacteria, **c** internal fungi, and **d** external fungi. Axes represent the two components of variation explaining the greatest proportion of variation in the data set. The third axis for each graph is 4.7%, 3.5%, 5.0%, and 8.0%, respectively. **e**–**h** Constrained analysis of principle coordinates on Bray-Curtis distances of **e** internal bacteria, **f** external bacteria, **g** internal fungi, and **h** external fungi. The variance explained is 4.37%, 6.86%, 4,18% and 6,00%, respectively, with *p* value of 0.001. The third axis for each graph is 21.3%, 19.3%, 13.6%, and 12.7%, respectively

Constrained analysis of principal coordinates (CAP) suggested that the sex of the flies only marginally explains the variance in the datasets, indicating that males and females do not have distinct bacterial or fungal microbiota (Table 1, Additional file 1: Figure S2D). In contrast, country, habitat, and site accounted for much more of the variance; habitat and sampling site accounted for 6.5% and 8.7% of the variance in external bacterial communities; and country accounted for 11% and 23% of the internal and external fungal communities, respectively (p ≤ 0.001, with significance determined by a permutation-based ANOVA test) (Table 1). A similar trend emerges from a principal coordinates analysis (PCoA) of Bray-Curtis (BC) distances (Fig. 2a–d). External bacterial, internal fungal, and external fungal communities clearly separated according to country, while the separation was weak for internal bacteria.

**Table 1:** Constrained analysis of principal coordinates of the bacterial and fungal community compositions

	Internal bacteria (397 samples)			External bacteria (294 samples)			Internal fungi (205 samples)			External fungi (112 samples)		
	% variance	*F*	*p*	% variance	*F*	*p*	% variance	*F*	*p*	% variance	*F*	*p*
Sex	0.4	1.5	0.016	0.6	1.7	0.006	0.8	1.6	0.050	1.0	1.2	0.260
Country	2.4	9.9	0.001	5.8	20.3	0.001	11.0	27.5	0.001	23.0	44.5	0.001
Country:habitat	4.0	4.1	0.001	6.5	5.6	0.001	3.5	2.2	0.001	7.2	2.8	0.001
Country:habitat:site	6.3	2.4	0.001	8.7	2.7	0.001	8.2	2.0	0.001	8.9	3.4	0.001

Using the CAP analysis and partialling out the “country” variable enabled to disentangle samples according to habitat (Fig. 2e–h). There was some clustering according to habitat for internal bacterial samples, but the separation was less marked than for the other microbial communities (Fig. 2e). External bacterial communities from each country separated distinctly with homes and hospitals clustering apart from farms (Fig. 2f). The fungal communities of the house flies also separated weakly based on the habitat, noticeably in the case of internal fly samples from Belgium, again with farms separating from hospitals and homes (Fig. 2g, h). These patterns were also observed when the data were collapsed at the genus level and the 100 most prevalent bacterial genera were clustered using PCoA with BC distances and samples were ordered by habitat (Additional file 1: Figure S3).

### Microbial community profiles of *M*. *domestica*

The distinct clustering of samples according to country and habitat led us to ask whether we could identify specific microbial populations for the different origins.

We first examined the most abundant bacterial and fungal classes associated with house flies sampled across the various habitats. The distribution of the most abundant bacterial classes was quite similar across the various habitats and included Bacilli, Gammaproteobacteria, and Actinobacteria (Additional file 1: Figure S4A). Notably, flies collected from the lab environment were distinct from the other samples and were strongly dominated by Gammaproteobacteria, especially on their external surface. Fungal classes Dothideomycetes, Eurotiomycetes, and Wallemiomycetes were found in flies from all habitat types (Additional file 1: Figure S4B), but the fungal communities were more varied, especially between countries. For example, there was a higher abundance of Wallemiomycetes in Belgian fly samples versus a higher abundance of Dothideomycetes in Rwandan fly samples.

**Fig. 3:**
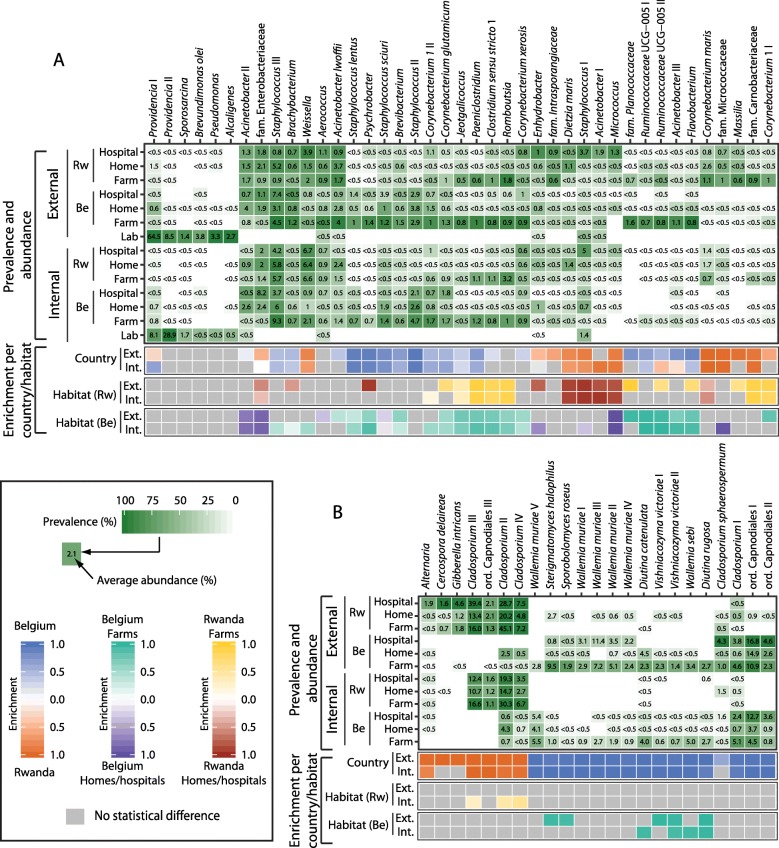
Microbial community profiles of *Musca domestica* microbiotas. **a** Bacterial and **b** fungal taxa were chosen to represent the most prevalent taxa in all habitats (present in > 50% of samples in each habitat separately, at 0.5% detection threshold—the resulting list was compiled, and unique ones chosen for analyses). The average abundance of the taxon in the habitat is given in the box as percentage. Prevalence heat map indicates the proportion of samples carrying each taxon in > 0.1% abundance. Enrichment of significantly different amplicon sequence variants (Kruskal-Wallis test, fdr corrected *p* value < 0.01) is indicated in the lower panels for internal and external samples: blue vs. orange = Belgium vs. Rwanda, turquoise vs. purple = farms vs. homes/hospitals in Belgium, yellow vs. red = farms vs. homes/hospitals in Rwanda. The bacterial community of the house flies shows distinct patterns of enrichment based on country and habitat, whereas the fungal community members are enriched mainly according to the flies’ country of origin

To identify a more detailed microbial signature for *M*. *domestica*, we analyzed the most prevalent ASVs from the habitats defined as being present in at least 50% of the samples in each habitat separately, at a 0.5% detection threshold. In accordance with the PCoA and CAP analyses, there was a clear division of the external bacterial communities based on habitat and of the fungal communities based on country (Fig. 3). Some ASVs were present in all habitat types, regardless of country, but others appeared to be country- or habitat-specific. For example, *Aerococcus* was prevalently found in all habitats (including lab-grown flies) while *Staphylococcus lentus*, *Psychrobacter*, and *Staphylococcus sciuri* were enriched in the samples from Belgium and *Weissella*, *Dietzia maris*, and *Micrococcus* were enriched in the flies from Rwanda.

Country-specific signatures were most clear in the fungal communities, with some ASVs found almost exclusively in one country or the other. For example, some ASVs corresponding to *Cladosporium* were found abundantly and prevalently in Rwandan samples (e.g., *Cladosporium* ASV III, with average abundance of 18.1% and average prevalence of 89.4%) but were absent from any Belgian sample. Conversely, several ASVs corresponding to Capnodiales and *Wallemia* were found in Belgium but were rarely found in Rwanda. The most abundant ASVs in Belgian habitats corresponded to Capnodiales ASV I, *Wallemia muriae* ASV III, and *Wallemia muriae* ASV V, with average abundance across Belgian habitats of 10.6%, 3.6%, and 3.0%, respectively, and prevalence of 80.4%, 21.0%, and 26.2%, respectively.

Habitat signatures were most clear in the bacterial communities, especially that of the farm habitat. *Jeotgalicoccus*, *Paeniclostridum*, *Clostridum sensu stricto 1*, and *Romboutsia* were enriched in the farm fly samples (both in Belgium and Rwanda), while *Micrococcus* was enriched in flies collected in homes and hospitals. Farm signatures were also dependent on the country. Planococcaceae, Ruminococcaceae, *Acinetobacter*, and *Flavobacterium* were significantly enriched in fly samples from Belgian farms whereas *Massilia*, Carnobacteriaceae, and *Corynebacterium* were enriched in samples from Rwandan farms. Moreover, all these taxa were more prevalent and abundant in the external samples than in the internal ones. There was also a distinct Rwandan hospital/home signature containing *Dietzia maris* (a potential human pathogen previously known as *Rhodococcus maris*), *Staphylococcus*, and *Acinetobacter* and a small grouping of *Acinetobacter* and *Enterobacteriaceae* enriched in samples from Belgian hospitals/homes. Overall, *Staphylococcus* ASV III was the most prevalent ASV in natural habitats, averaging at 80% prevalence, followed by *Weissella* with average prevalence of 64% across habitats. The overall abundances of the ASVs in natural habitats were rather low, with *Staphylococcus* ASV III having the highest value (4.7%). Overall, the majority of the abundant taxa were present both in internal and external compartments of the flies with similar abundances (Additional file 1: Figure S5). However, some ASVs corresponding to Planococcaceae, *Pseudomonas bauzanensis*, and Ruminococcaceae were more abundant on the external surface, while other ASVs corresponding to *Acinetobacter*, *Nocardiopsis prasina*, and Lactobacillales were more abundant in the internal microbiota. Further, lab flies displayed a distinct bacterial community profile compared to flies from environmental habitats. Notably, *Providencia* is highly abundant and prevalent but taxa from other habitats were found at relatively low abundance or were completely absent in the lab flies (Fig. 3a).

## Discussion

The microbiota of an organism is shaped by a range of complex factors [19]. For insects, the microbiota is mainly influenced by developmental stage, physiochemical conditions in different gut compartments, accessible sources for acquisition of microbes (such as food and habitat), and the transfer of microorganisms to offspring [18]. To our knowledge, this study represents the first large-scale culture-independent study investigating both the bacterial and fungal communities associated with *M*. *domestica* sampled at different locations. The data therefore do not only offer an in-depth view of the microbial diversity, but also yield insight into the influence of the habitat. Our results reveal that house flies harbor a very diverse microbiota, which is influenced by habitat and geographical origin, especially for the bacterial portion of the external microbiota, and the fungal part of both the external and internal microbiota.

Interestingly, the overall internal bacterial communities proved to be relatively similar, regardless of country or habitat (Figs. 1a and 2a, e; Additional file 1: Figure S2A; Fig. 3a). This finding is in accordance with a previous study that found that the internal bacterial community composition was consistent across house flies sampled from various farms in Denmark [7]. This suggests a relatively stable internal bacterial community composition that is likely influenced by the fly physiology and less so by the environment. In addition, vertical transmission of the bacteria from parent to offspring can further consolidate regional differences in microbiota. There is evidence that some symbiotic bacteria such as *Klebsiella oxytoca* are transferred onto the fly eggs during oviposition and maintained throughout the insect life cycle [20, 21]. Using the V4 region of the 16S rRNA gene, taxa belonging to *Klebsiella* can only be accurately identified to the family level (*Enterobacteriaceae*), which was prevalently observed in the fly samples of this study [22].

In contrast to the internal microbiota, the external surface of the house flies carried a specific bacterial signature, especially when the sampling location has abundant sources of bacteria—such as the farm habitat (Fig. 3a) [23]. Previous studies which sampled microbes from a cowshed environment, found similar bacterial communities, including Ruminococcaceae members which were also distinctly enriched in fly samples from farms compared to hospitals and homes in this study [24, 25] (Fig. 3). Recent years have seen increased interest in the microbiome of human-constructed environments, and several studies have sampled the air and surfaces in various buildings including hospitals [26, 27]. These studies have revealed an abundance of bacteria associated with human skin and oral sites, including *Staphylococcus*, *Streptococcus*, and *Corynebacterium*, suggesting that humans are a major source of microbes found in built environments [26–28]. Interestingly, these genera were also notably abundant in the fly samples investigated in this study (Fig. 3, Additional file 1: Figure S3AB), indicating that some part of the house fly microbiome might be acquired from human skin, either through direct contact or indirectly by sharing the same environment. The bacterial habitat signatures were the strongest in the external compartment samples, indicating that to fully understand the vectoring potential of insects, the external surface should be investigated thoroughly (Fig. 3a). Feeding and reproduction habits of house flies involve microbe-rich substrates; therefore, it is not surprising that the external surface picks up a variety of microbes [6].

The observed diversity of fungi was distinctly lower than that of bacteria and dominated by molds from the genera *Cladosporium* and *Wallemia*. Fungi from genus *Cladosporium* were predominant in flies sampled in Rwanda, whereas members of the order Capnodiales and the genus *Wallemia* were more prominent in Belgian fly samples (Fig. 3b). The differences between flies’ fungal communities were clearly driven by sampling country and less by habitat. This observation remained evident as well on the class level (Additional file 1: Figure S4B). Members of the genus *Cladosporium* are very diverse (772 species) and are found world-wide in air, soil, and plant debris [29, 30]. Additionally, *Cladosporium* has been frequently isolated from house flies [16, 31]. By contrast, here, for the first time, *Wallemia* is linked to *M*. *domestica*. This is possibly due to the fact that previous studies investigating the fungal communities of house flies used culture-dependent methods, while *Wallemia* is known to be difficult to cultivate [32]. Members of the genus *Wallemia* are coping well with stressful environmental conditions (e.g., low availability of water and high-salinity) and include air-born food-contaminants capable of causing health issues for humans (allergies, asthma) [33, 34]. *Cladosporium* genus contains several entomopathogenic species and *Wallemia* have been shown to be fatal to some insects [35–37]. Moreover, insects can be also attracted to the volatile compounds from *Cladosporium* signaling suitable food sources or oviposition sites [38, 39]. Whether the fungi observed in this study have a relevant role for the house fly or they are present solely due to environmental exposure remains to be investigated [40]. The clear separation of fungal ASVs based on the house flies’ country of origin was rather surprising given that the bacterial ASVs overlapped considerably based on country. Belgium and Rwanda differ in climate and are located at distinct geographic locations. This might indicate that the fungal portion of the house fly microbiota is highly dependent on the influx from the environment and less restricted by the physiology of the insects.

Data obtained from lab-grown flies further confirm the importance of the environment in establishing the fly microbiota. Lab-grown flies showed lower microbial loads, with only four flies yielding sufficient internal bacterial DNA and six specimens sufficient external bacterial DNA for the analyses to be performed. Moreover, the overall microbial diversity of lab-grown flies was rather low compared to wild flies (Fig. 1, Additional file 2: Table S1). This enforces the hypothesis that most of the house fly’s microbiota is acquired from the environment initially, even if some of the potential symbiotic species are passed on to offspring, with the circle being interrupted in case of a house fly lab strain. Furthermore, the low microbial colonization of lab flies could indicate that *M*. *domestica* can survive with very low amounts of bacteria in their gut, contrary to many other insects, yet comparable with the caterpillars shown to be lacking a resident gut microbiome [41, 42]. While in nature, the adult flies live between 15 and 25 days and can survive up to 2 months, the lab-grown flies were captured during 24 h after emergence from pupae [43]. It has been shown in fruit flies that after mid-life, the flies’ intestinal barrier becomes dysfunctional leading to age-related changes of the microbiota [44]. Similar studies are absent in house flies, but the age disparity between the flies sampled in labs and in natural habitats could impact the composition of the flies’ microbiota. The bacterial composition identified in the lab-grown flies was dominated by members of the genus *Providencia* which has been previously observed in lab-grown *M*. *domestica* larvae [45]. Members of the genus *Providencia* have been isolated from a range of environments and organisms and include among others opportunistic pathogens of humans and insects [46, 47].

Interestingly, several ASVs lacked lower level taxonomic identification, which could indicate the presence of a number of new species. Indeed, in the framework of this study, we identified a novel prevalent bacterial species, for which the name *Apibacter muscae* was proposed (Additional file 1: Figure S3A,B) [48]. The *Apibacter* genus has been recently described as a novel genus, with several strains from bee species and it appears to include various insect-associated bacterial species [49, 50]. It may be expected that other novel species will be described from the fly’s microbiome in the near future.

Many of the prevalent genera identified in this study, such as *Dietzia*, *Providencia*, *Pseudomonas Staphylococcus*, *Acinetobacter*, and *Micrococcus*, as well the fungi from the genera *Alternaria* and *Wallemia*, are known to contain potentially pathogenic species of clinical relevance. These findings are in accordance with previous observations of pathogenic microbes in house flies, recently reviewed in Khamesipour et al [16]. Several genera listed in this review were also found in this study, with some exhibiting location-specific patterns. The genera *Streptococcus* and *Micrococcus* were more prevalent in hospital fly samples whereas *Clostridium* and *Escherichia-Shigella* were more prevalent in farm fly samples (Additional file 3: Table S3). Interestingly, the ASVs corresponding to *Streptococcus pyogenes*, known to cause a range of human diseases, were highly abundant in hospital samples, while being absent in farm and home samples [51]. One could speculate that house flies are helping to spread pathogenic *Streptococci* and *Micrococci* in the hospital environments; however, the investigated 16S rRNA gene marker does not provide sufficient resolution for identification down to species and strain level to make firm conclusions regarding the virulence of these microbes [52].

Altogether, our results reveal a species-rich house fly microbiota, with specific bacterial species found internally independent of fly origin, while the microbial community composition on the body surface varies more with geographical location and habitat. Among others, several genera with potentially pathogenic species were found to be carried by the house flies. Moreover, we have shown that microbes from the environment readily associate with the outer surface of the flies, especially in habitats rich in decaying and fecal matter, such as farms. Therefore, this study reinforces the concept that during disease outbreaks, when pathogens are prevalent, house flies can be important vectors [53].

## Conclusions

Together, our results show that *M*. *domestica* is associated with a highly diverse microbiota. While the internal bacterial community is relatively similar between flies from different sampling locations, the external bacterial communities are influenced by the country of origin and, even more so, by the habitat of the fly. This suggests that the outer surface may be most important for vectoring a broad spectrum of environmental as well as pathogenic microbes, while the inner body is more restricted to microbes that survive in these conditions and may act as fly symbionts. For fungi, however, both the internal and external populations varied with country and habitat, suggesting that the identified fungi may represent transient microorganisms rather than commensals. Further research is needed to elucidate the possible functions of these microbes, their original source, and transmission patterns.

## Methods

### Sample collection

*Musca domestica* adults were collected from sites near Brussels, Belgium, and Butare, Rwanda, between March and October 2017 (Additional file 4: Table S4). Additionally, eight flies were collected in August to October 2016. In total, over 400 flies were collected from three cow farms, four homes, and one hospital in Belgium and three cow farms, three homes, and three hospitals in Rwanda (Additional file 1: Figure S1A). The Rwandan hospital samples were mainly from patient rooms and waiting rooms, while in the Belgian hospital the flies were mainly found in corridors except for one fly in a patient room. On average, eight males and ten females were analyzed from each site in Belgium, and 15 males and 14 females in Rwanda. In addition, 15 male and 15 female lab-grown World Health Organization WHOij2 strain flies were captured within 24 h post-emergence and included in the study.

Individual flies were directly caught into sterile 50-mL Falcon tubes and immediately put on ice and stored at − 20 °C. Flies were photographed to document the sex (based on the width between eyes) and transferred to sterile 1.5-ml microcentrifuge tubes (one per tube) and further stored at − 20 °C. Flies from Rwanda were shipped to the laboratory in Leuven, Belgium, on dry ice before further storage at − 20 °C. Upon defrosting, flies were washed with 230 μl of phosphate-buffered saline with 0.01% Tween80 (PBS-T) by gently vortexing for 40 s (Additional file 1: Figure S1C). Subsequently, the washing solution was used as an “external sample.” Additionally, flies were sterilized with 2.5% NaOCl (VWR) and subsequently washed twice with PBS-T. Each fly was then homogenized in 450 μl PBS-T with a motorized homogenizer (Cordless Pestle Motor, VWR), to obtain “internal” samples. The PBS-T was prepared once and frozen in aliquots to be used throughout the study.

### DNA extraction and amplicon sequencing

Genomic DNA was isolated from all external (fly washing solution) and internal (whole-fly homogenate) samples using the Qiagen RNeasy PowerMicrobiome Kit according to the manufacturer’s protocols with the exception of replacing the β-mercaptoethanol with Tris (2-carboxyethyl) phosphine hydrochloride (0.01 M, Sigma). Additionally, in every extraction batch, a negative control was included in which clean PBS-T was used as the starting material. Each internal sample was also used for fly species confirmation by sequencing the cytochrome oxidase I gene (primers LCO1490/ HCO2198 and LepF1/ LepR1 [54–56]).

Illumina barcoded primers, designed according to Kozich et al. [57], were used to amplify the V4 region of the bacterial 16S rRNA gene (primers 515F and 806R) or the fungal ITS2 region (primers ITS86F and ITS4) [57] (Additional file 5: Table S5). Triplicate 25-μl reactions were run for 34 cycles of amplification consisting of 45 s at 95 °C, 45 s at 58 °C, and 45 s at 72 °C using Titanium Taq DNA polymerase (Clontech). The extraction blanks and negative PCR controls, where template was replaced by Microbial DNA-Free Water (Qiagen), were amplified alongside the fly samples. In addition, two mock community DNA samples were included, one for bacteria and one for fungi. Mixed genomic DNA from a bacterial mock community (mock community A; HM-278D; even, low concentration of each bacterial species) was provided by BEI Resources (Additional file 3: Table S6). Further, a fungal mock community was made in house by growing different species separately in liquid YPD (yeast extract peptone glucose), counting the cells and pooling equal numbers of cells from each species prior to DNA extraction (Additional file 3: Table S6). PCR products from triplicate PCR runs were combined, analyzed with the QIAxcel Advanced fragment analyzer (Qiagen), and samples with an amplicon concentration above 1.5 nM were pooled to an equimolar concentration. PCR products from blank samples and PCR negative controls were added in constant 60-μl volume. The pooled amplicons were purified using QIAquick PCR Purification Kit (Qiagen). BluePippin Size Selection (Sage Science) was performed to remove any unspecific DNA (selecting for amplicons in the range of 320–500 bp for V4 and 250–1000 bp for ITS2). With the exception of the eight flies collected in 2016 and sequenced earlier following the same protocol, the rest of the samples were sequenced in five runs on an Illumina MiSeq platform (three runs of 2 × 250 bp for bacterial samples and two runs of 2 × 300 bp for fungal samples) at the VIB Nucleomics Core facility. Each run included the respective bacterial or fungal mock community sample. The taxa distribution of bacterial and fungal mock communities demonstrated that the experimental conditions were met to achieve robust data and that the results between different sequencing runs were comparable (Additional file 1: Figure S6).

### Bioinformatic processing

Demultiplexed reads were processed with the QIIME2 (v 2018.11) pipeline using the various built-in plugins cited below [58]. The primers were removed from the reads using cutadapt (v 2018.11.0) and the ITS2 of the fungal reads was extracted from the reads using itsxpress (v 1.7.2) [59, 60]. The reads from each run were separately quality filtered and merged using the DADA2 (v 2018.11.0) algorithm [61]. Resulting ASV tables and representative sequences were merged subsequently, while keeping the bacterial and fungal data sets separate. The reads were classified using the feature-classifier plugin from QIIME2 with the classify-sklearn method [62, 63]. The databases used for taxonomic assignment were Silva 132 SSU Ref NR 99 for bacteria and UNITE version 7.2 for fungi [64, 65]. The data sets were then exported from QIIME2 and analyzed in R (v3.5.2) with package decontam (v1.2.0) to determine the contaminants based on the ASV prevalence in true samples versus the blank samples and negative PCR controls [66, 67]. The following ASVs were thereafter filtered from the data sets: ASVs determined as contaminants with the decontam package, sequences classified as mitochondria or chloroplasts, ASVs with the kingdom level assignment “Eukaryota” or “Archaea” and ASVs without kingdom (and phylum level for bacteria) assignment (accounting for a low amount of spurious reads). Samples with less than 3000 reads as well as ASVs representing singletons and doubletons were removed from the further analyses.

### Statistical analysis

Alpha diversities were calculated on rarified data set (3000 reads for bacteria and 4000 reads for fungi) using QIIME2 pipeline. The statistical comparisons of alpha diversities between external and internal compartments were done using Kruskal-Wallis tests and *p* values adjusted with Benjamini-Hochberg correction. The succeeding analyses were performed on relative abundance data sets in R (v3.5.2). The CAP analysis (Table 1) was performed using the capscale function from vegan package (v2.5-4) with the formula capscale (formula = DistBC ~ Sex + Condition(Miseqrun+ Date_homogenized + Extraction_kit_batch + Extraction_batch+ Date_DNA_Extr), data = design) and capscale (formula = DistBC ~ Country/Habitat/Site + Condition(Miseqrun+ Date_homogenized + Extraction_kit_batch + Extraction_batch+ Date_DNA_Extr), data = design) where the “DistBC” denotes the Bray-Curtis dissimilarity matrix, the “design” is the metadata table and the constrained items are biological variables, conditioned with the technical variables [68]. The variable “Sex” was analyzed separately as it is independent of other variables, while the variables “Country, Habitat and Site” are nested in one another. The significance of the constraints was thereafter estimated with a permutation test using anova.cca function from vegan package (v2.5-4). The PCoA plots (Fig. 2a–d) were generated from the Bray-Curtis dissimilarity matrix with the plot_ordination function from phyloseq package (v1.26.0) [69]. The CAP plots (Fig. 2e–h) were generated using the capscale function (capscale(DistBC ~ design$Habitat + Condition(design$Country))) and cpcoa.func.R published by Zgadzaj et al. at http://www.mpipz.mpg.de/R_scripts [70]. Plot_heatmap function from phyloseq package (v1.26.0) was used to organize the 100 most abundant genera based on PCoA ordination with Bray-Curtis dissimilarity matrix (Additional file 1: Figure S3) [71]. The prevalent ASVs per habitat were defined using the core function from the microbiome package (v1.4.2) with detection limit set at 0.5% and prevalence threshold to above 50% [72]. The enrichment of the ASVs by country or habitat was tested using Kruskal-Wallis test and *p* values adjusted with fdr method. For ASVs that were determined statistically different with *p* value under 0.01, the enrichment was calculated with formula (*x* − *y*)/*x*, where *x* and *y* are the average abundances of the given ASV in the environments that are compared, with *x* the environment where the ASV is enriched. Visualizations were done using phyloseq (v1.26.0) and ggplot2 (v3.1.0) packages [69, 73].

## Supplementary information

**Additional file 1: Figure S1.** House flies were collected from two geographically distinct locations and three different habitats. (A) Sampling locations were located near Brussels, Belgium and Butare, Rwanda (dotted box indicates zoomed in region in each panel). (B) Geographical coordinates of the sampling sites from each country. (C) Individual flies were washed with PBS-Tween80 (external sample) and subsequently homogenized (internal sample). Both samples were subjected to amplicon sequencing. **Figure S2.** Venn diagrams of ASVs shared between flies external and internal compartment (A), samples from Belgium and Rwanda (B), from different environments (C) and the two sexes (D). ASVs present in each category with prevalence of >10% at detection threshold of 0.1% were included in the comparisons. **Figure S3.** Heatmap of 100 most abundant genera of the (A) internal bacterial (B) external bacterial (C) internal fungal and (D) external fungal microbiotas depicting the clustering of taxa based on PCoA ordination with Bray-Curtis distances and samples by habitat. Panel A (internal bacteria) does not show a clear clustering of species with the location where the flies were caught whereas Panel B (external bacteria) shows weak clustering and Panels C (internal fungi) and D (external fungi) show a clear separation of microbial genera isolated from flies caught at different locations. **Figure S4.** Relative abundance of bacterial (A) and fungal (B) classes, grouped by habitat. The classes with overall average relative abundance under 1% are grouped in “Other”. **Figure S5.** Comparison of average relative abundance for most abundant ASVs across internal and external house fly bacterial (A) and fungal (B) microbiota. Dashed lines indicate 1% of the average microbiota in external and internal compartments (log10 scale). The plots include the 1000 most abundant ASVs from internal and the 1000 most abundant ASVs from external samples. For bacteria, the ASVs above 1% in either internal or external compartment are labelled, as well as ASVs that are above 0.25% abundance in one compartment and less than 0.04% in the other compartment. For fungi, the ASVs above 2.5% in either internal or external compartment are labelled, as well as ASVs that are above 1% abundance in one compartment and less than 0.003% in the other compartment. **Figure S6.** The bacterial (A) and fungal (B) mock communities were sequenced along with fly samples in each sequencing run and juxtaposed with the true (‘Expected’) composition and abundance of the mock community.

**Additional file 2: Table S1.** Overview of sampling success rate.

**Additional file 3: Table S2.** Alpha diversity measured by Shannon index and observed amplicon sequence variants (ASVs) of the grouped samples. **Table S3.** The prevalence and abundance of pathogenic bacteria from house flies displaying location specific patterns. Initial analysis and selection was based on Table 1 in Khamesipour et al 2018 [16] enlisting main pathogenic bacterial genera and species observed in house flies. ** Table S6.** Composition of the mock communities.

**Additional file 4: Table S4.** Metadata of the samples..

**Additional file 5: Table S5.** Sequencing primers.

## Data Availability

The sequences of 16S rRNA gene and ITS were deposited in the Sequence Read Archive (SRA) at NCBI under Bioproject PRJNA544040.

## Supplementary information

**Supplementary information** accompanies this paper at 10.1186/s40168-019-0748-9 and on Dryad repository 10.5061/dryad.cj212m6.
