# Supplementary material for: Microbial communities of the house fly Musca domestica vary with geographical location and habitat
Source: Microbiome. 2019 Nov 8;7:147. doi: 10.1186/s40168-019-0748-9 (PMC6839111; doi:10.1186/s40168-019-0748-9)
Supplement: Supplementary file 1 — Additional file 1: Figure S1. House flies were collected from two geographically distinct locations and three different habitats. (A) Sampling locations were located near Brussels, Belgium and Butare, Rwanda (dotted box indicates zoomed in region in each panel). (B) Geographical coordinates of the sampling sites from each country. (C) Individual flies were washed with PBS-Tween80 (external sample) and subsequently homogenized (internal sample). Both samples were subjected to amplicon sequencing. Figure S2. Venn diagrams of ASVs shared between flies external and internal compartment (A), samples from Belgium and Rwanda (B), from different environments (C) and the two sexes (D). ASVs present in each category with prevalence of >10% at detection threshold of 0.1% were included in the comparisons. Figure S3. Heatmap of 100 most abundant genera of the (A) internal bacterial (B) external bacterial (C) internal fungal and (D) external fungal microbiotas depicting the clustering of taxa based on PCoA ordination with Bray-Curtis distances and samples by habitat. Panel A (internal bacteria) does not show a clear clustering of species with the location where the flies were caught whereas Panel B (external bacteria) shows weak clustering and Panels C (internal fungi) and D (external fungi) show a clear separation of microbial genera isolated from flies caught at different locations. Figure S4. Relative abundance of bacterial (A) and fungal (B) classes, grouped by habitat. The classes with overall average relative abundance under 1% are grouped in “Other”. Figure S5. Comparison of average relative abundance for most abundant ASVs across internal and external house fly bacterial (A) and fungal (B) microbiota. Dashed lines indicate 1% of the average microbiota in external and internal compartments (log10 scale). The plots include the 1000 most abundant ASVs from internal and the 1000 most abundant ASVs from external samples. For bacteria, the ASVs above 1% in either internal or ex [file 40168_2019_748_MOESM1_ESM.pdf]

Figure S1

A

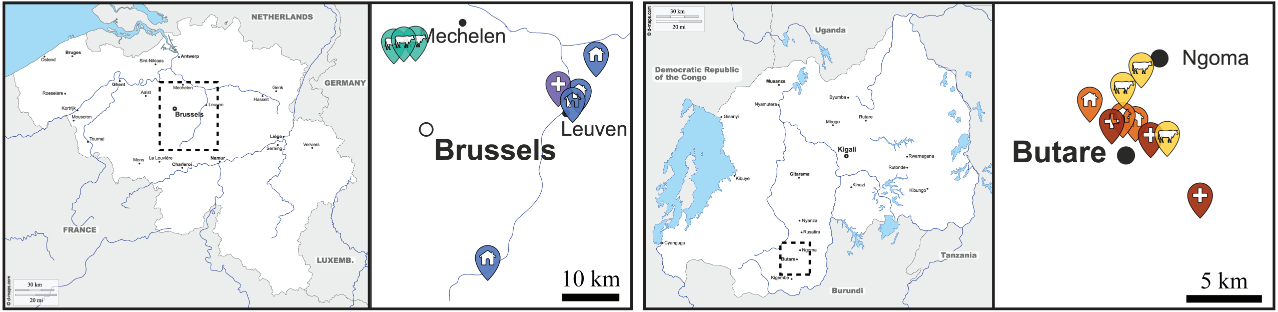

B

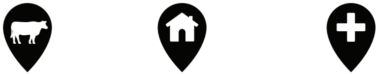

	Farms	Homes	Hospitals
**Belgium**	50.991972, 4.282715<br>50.991715, 4.279653<br>50.990618, 4.289008	50.866309, 4.710887<br>50.880713, 4.718678<br>50.785502, 4.588548<br>50.986216, 4.836012	50.878240, 4.672927
**Rwanda**	-2.586192, 29.752107<br>-2.605701, 29.753504<br>-2.562347, 29.761564	-2.603652, 29.742447<br>-2.593389, 29.740028<br>-2.602376, 29.742477	-2.607050, 29.747836<br>-2.648569, 29.783236<br>-2.604755, 29.741463

C

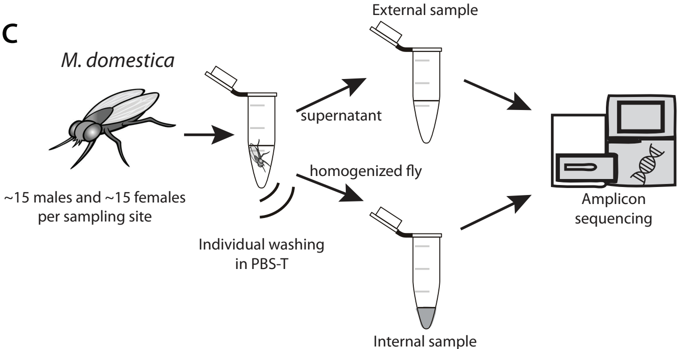

Figure S2

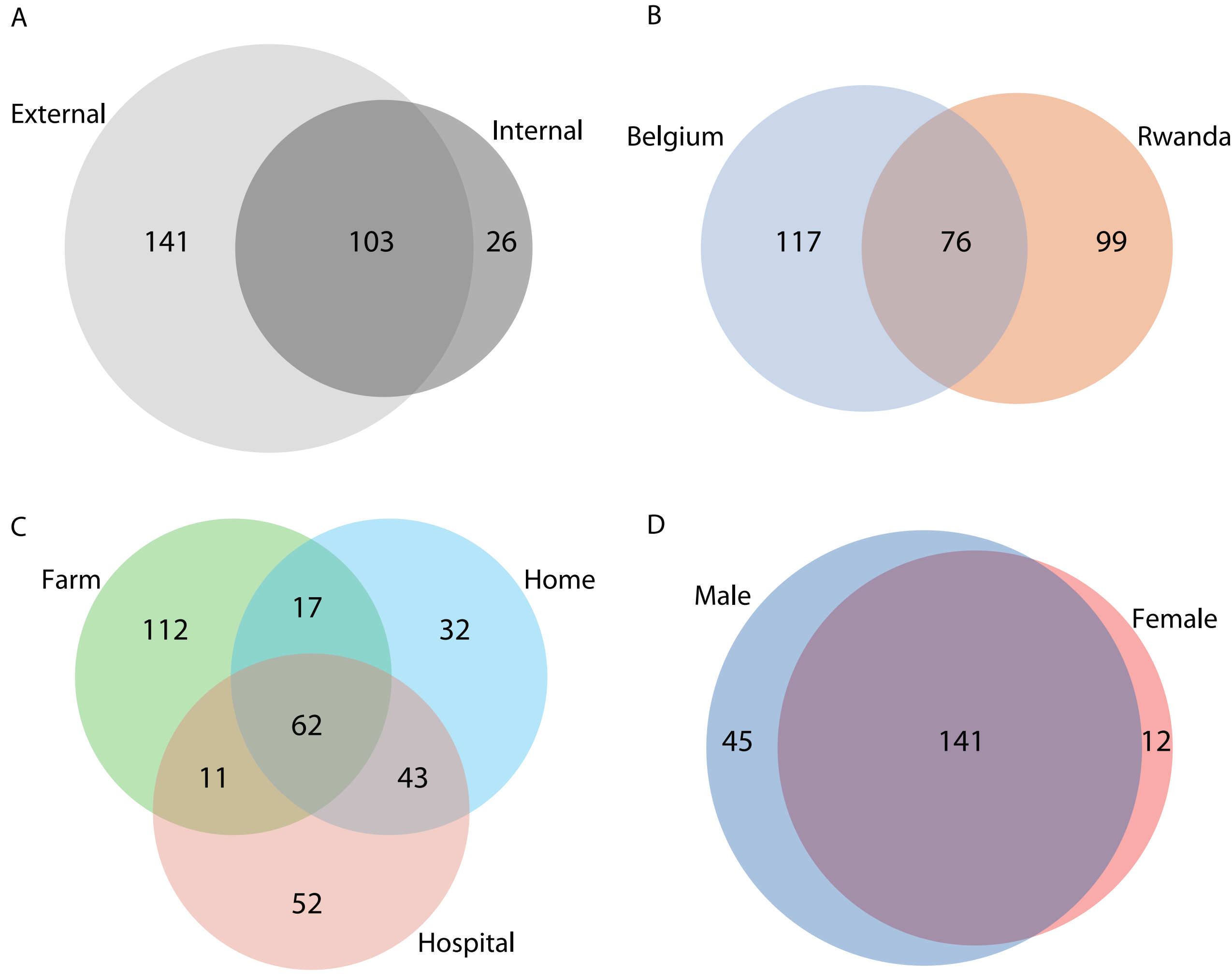

A
External
Internal
141
103
26
B
Belgium
Rwanda
117
76
99
C
Farm
Home
Hospital
112
17
32
62
11
43
52
D
Male
Female
45
141
12

Figure S3

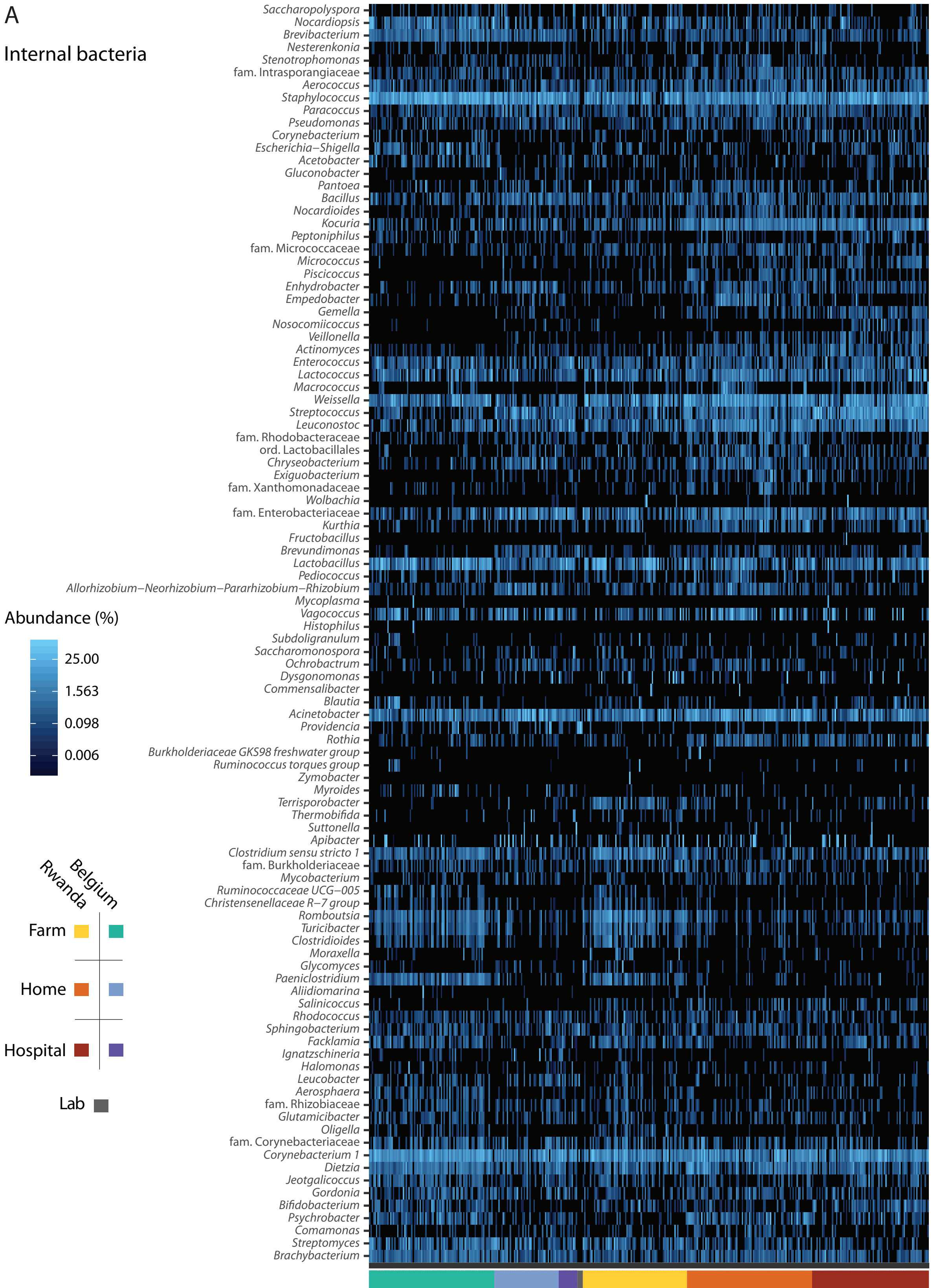

A
Internal bacteria
Saccharopolyspora
Nocardiopsis
Brevibacterium
Nesterenkonia
Stenotrophomonas
fam. Intrasporangiaceae
Aerococcus
Staphylococcus
Paracoccus
Pseudomonas
Corynebacterium
Escherichia–Shigella
Acetobacter
Gluconobacter
Pantoea
Bacillus
Nocardioides
Kocuria
Peptoniphilus
fam. Micrococcaceae
Micrococcus
Piscicoccus
Enhydrobacter
Empedobacter
Gemella
Nosocomiicoccus
Veillonella
Actinomyces
Enterococcus
Lactococcus
Macrococcus
Weissella
Streptococcus
Leuconostoc
fam. Rhodobacteraceae
ord. Lactobacillales
Chryseobacterium
Exiguobacterium
fam. Xanthomonadaceae
Wolbachia
fam. Enterobacteriaceae
Kurthia
Fructobacillus
Brevundimonas
Lactobacillus
Pediococcus
Allorhizobium–Neorhizobium–Pararhizobium–Rhizobium
Mycoplasma
Vagococcus
Histophilus
Subdoligranulum
Saccharomonospora
Ochrobactrum
Dysgonomonas
Commensalibacter
Blautia
Acinetobacter
Providencia
Rothia
Burkholderiaceae GKS98 freshwater group
Ruminococcus torques group
Zymobacter
Myroides
Terrisporobacter
Thermobifida
Suttonella
Apibacter
Clostridium sensu stricto 1
fam. Burkholderiaceae
Mycobacterium
Ruminococcaceae UCG–005
Christensenellaceae R–7 group
Romboutsia
Turicibacter
Clostridioides
Moraxella
Glycomyces
Paeniclostridium
Aliidiomarina
Salinicoccus
Rhodococcus
Sphingobacterium
Facklamia
Ignatzschineria
Halomonas
Leucobacter
Aerosphaera
fam. Rhizobiaceae
Glutamicibacter
Oligella
fam. Corynebacteriaceae
Corynebacterium 1
Dietzia
Jeotgalicoccus
Gordonia
Bifidobacterium
Psychrobacter
Comamonas
Streptomyces
Brachybacterium
Abundance (%)
25.00
1.563
0.098
0.006
Rwanda
Belgium
Farm
Home
Hospital
Lab

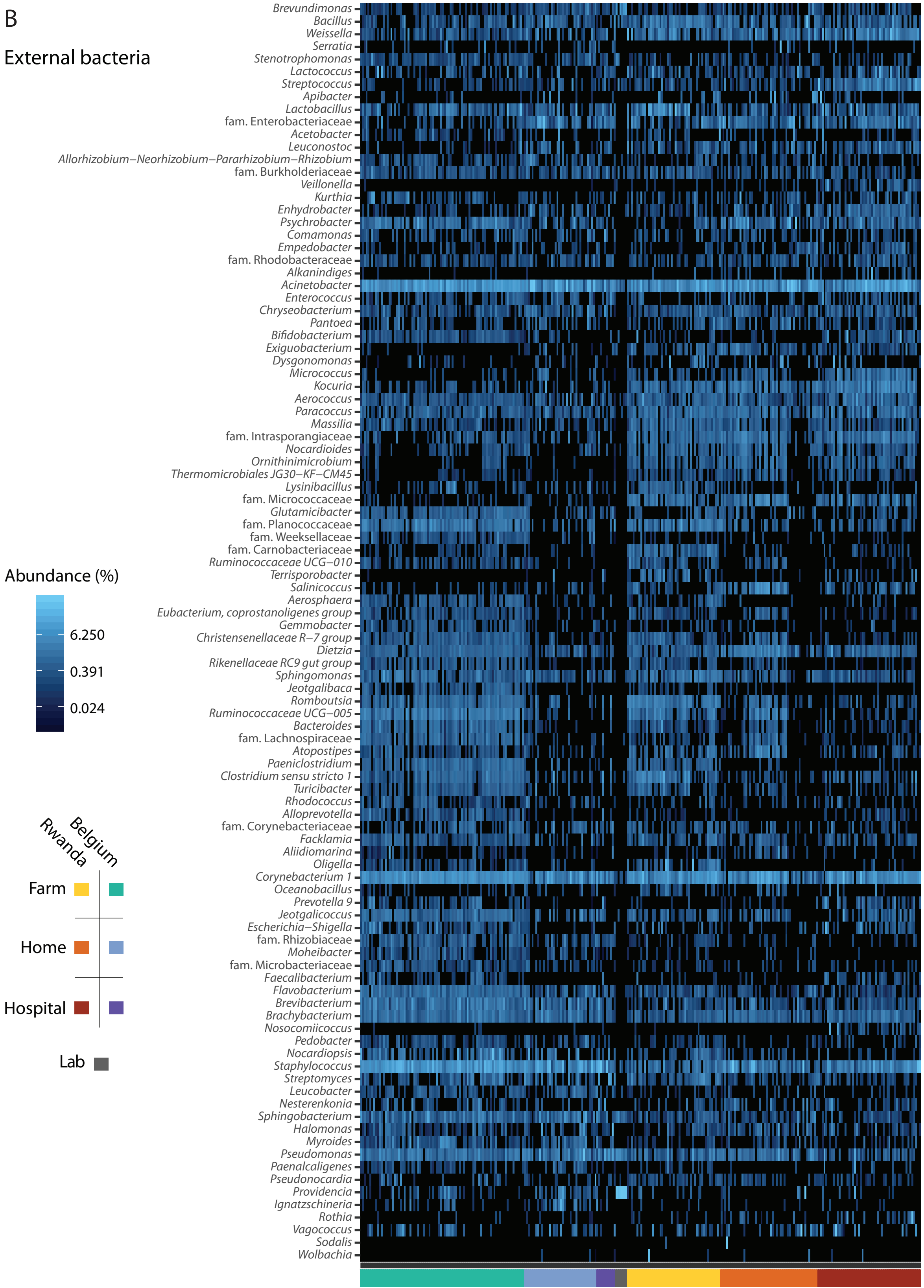

B
External bacteria
Brevundimonas
Bacillus
Weissella
Serratia
Stenotrophomonas
Lactococcus
Streptococcus
Apibacter
Lactobacillus
fam. Enterobacteriaceae
Acetobacter
Leuconostoc
Allorhizobium–Neorhizobium–Pararhizobium–Rhizobium
fam. Burkholderiaceae
Veillonella
Kurthia
Enhydrobacter
Psychrobacter
Comamonas
Empedobacter
fam. Rhodobacteraceae
Alkanindiges
Acinetobacter
Enterococcus
Chryseobacterium
Pantoea
Bifidobacterium
Exiguobacterium
Dysgonomonas
Micrococcus
Kocuria
Aerococcus
Paracoccus
Massilia
fam. Intrasporangiaceae
Nocardioides
Ornithinimicrobium
Thermomicrobiales JG30–KF–CM45
Lysinibacillus
fam. Micrococcaceae
Glutamicibacter
fam. Planococcaceae
fam. Weeksellaceae
fam. Carnobacteriaceae
Ruminococcaceae UCG–010
Terrisporobacter
Salinicoccus
Aerosphaera
Eubacterium, coprostanoligenes group
Gemmobacter
Christensenellaceae R–7 group
Dietzia
Rikenellaceae RC9 gut group
Sphingomonas
Jeotgalibaca
Romboutsia
Ruminococcaceae UCG–005
Bacteroides
fam. Lachnospiraceae
Atopostipes
Paeniclostridium
Clostridium sensu stricto 1
Turicibacter
Rhodococcus
Alloprevotella
fam. Corynebacteriaceae
Facklamia
Aliidiomarina
Oligella
Corynebacterium 1
Oceanobacillus
Prevotella 9
Jeotgalicoccus
Escherichia–Shigella
fam. Rhizobiaceae
Moheibacter
fam. Microbacteriaceae
Faecalibacterium
Flavobacterium
Brevibacterium
Brachybacterium
Nosocomiicoccus
Pedobacter
Nocardiopsis
Staphylococcus
Streptomyces
Leucobacter
Nesterenkonia
Sphingobacterium
Halomonas
Myroides
Pseudomonas
Paenalcaligenes
Pseudonocardia
Providencia
Ignatzschineria
Rothia
Vagococcus
Sodalis
Wolbachia
Abundance (%)
6.250
0.391
0.024
Rwanda
Belgium
Farm
Home
Hospital
Lab

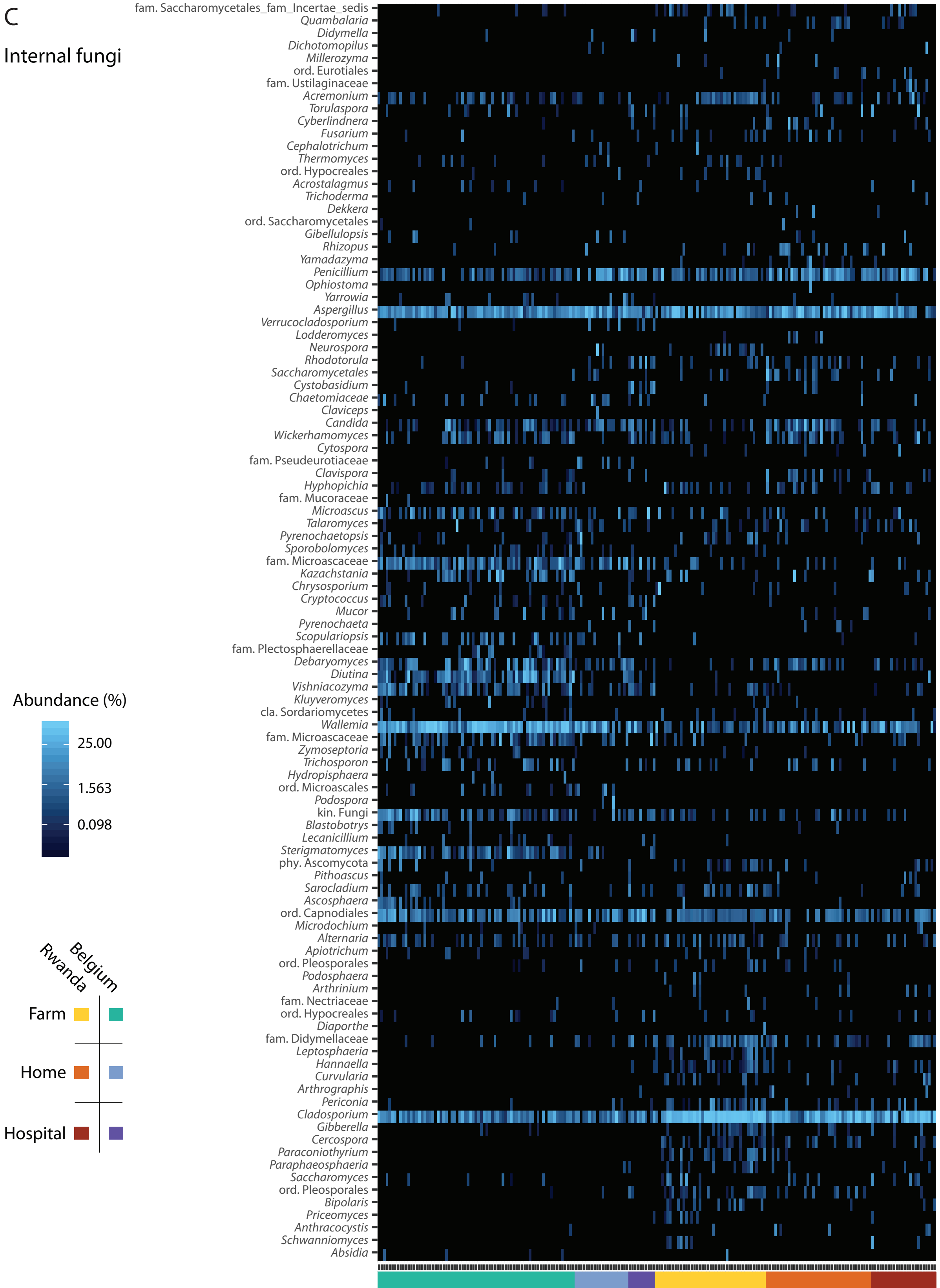
C
Internal fungi
fam. Saccharomycetales_fam_Incertae_sedis
Quambalaria
Didymella
Dichotomopilus
Millerozyma
ord. Eurotiales
fam. Ustilaginaceae
Acremonium
Torulaspora
Cyberlindnera
Fusarium
Cephalotrichum
Thermomyces
ord. Hypocreales
Acrostalagmus
Trichoderma
Dekkera
ord. Saccharomycetales
Gibellulopsis
Rhizopus
Yamadazyma
Penicillium
Ophiostoma
Yarrowia
Aspergillus
Verrucocladosporium
Lodderomyces
Neurospora
Rhodotorula
Saccharomycetales
Cystobasidium
Chaetomiaceae
Claviceps
Candida
Wickerhamomyces
Cytospora
fam. Pseudeurotiaceae
Clavispora
Hyphopichia
fam. Mucoraceae
Microascus
Talaromyces
Pyrenochaetopsis
Sporobolomyces
fam. Microascaceae
Kazachstania
Chrysosporium
Cryptococcus
Mucor
Pyrenochaeta
Scopulariopsis
fam. Plectosphaerellaceae
Debaryomyces
Diutina
Vishniacozyma
Kluyveromyces
cla. Sordariomycetes
Wallemia
fam. Microascaceae
Zymoseptoria
Trichosporon
Hydropisphaera
ord. Microascales
Podospora
kin. Fungi
Blastobotrys
Lecanicillium
Sterigmatomyces
phy. Ascomycota
Pithoascus
Sarocladium
Ascosphaera
ord. Capnodiales
Microdochium
Alternaria
Apiotrichum
ord. Pleosporales
Podosphaera
Arthrinium
fam. Nectriaceae
ord. Hypocreales
Diaporthe
fam. Didymellaceae
Leptosphaeria
Hannaella
Curvularia
Arthrographis
Periconia
Cladosporium
Gibberella
Cercospora
Paraconiothyrium
Paraphaeosphaeria
Saccharomyces
ord. Pleosporales
Bipolaris
Priceomyces
Anthracocystis
Schwanniomyces
Absidia
Abundance (%)
25.00
1.563
0.098
Rwanda
Belgium
Farm
Home
Hospital

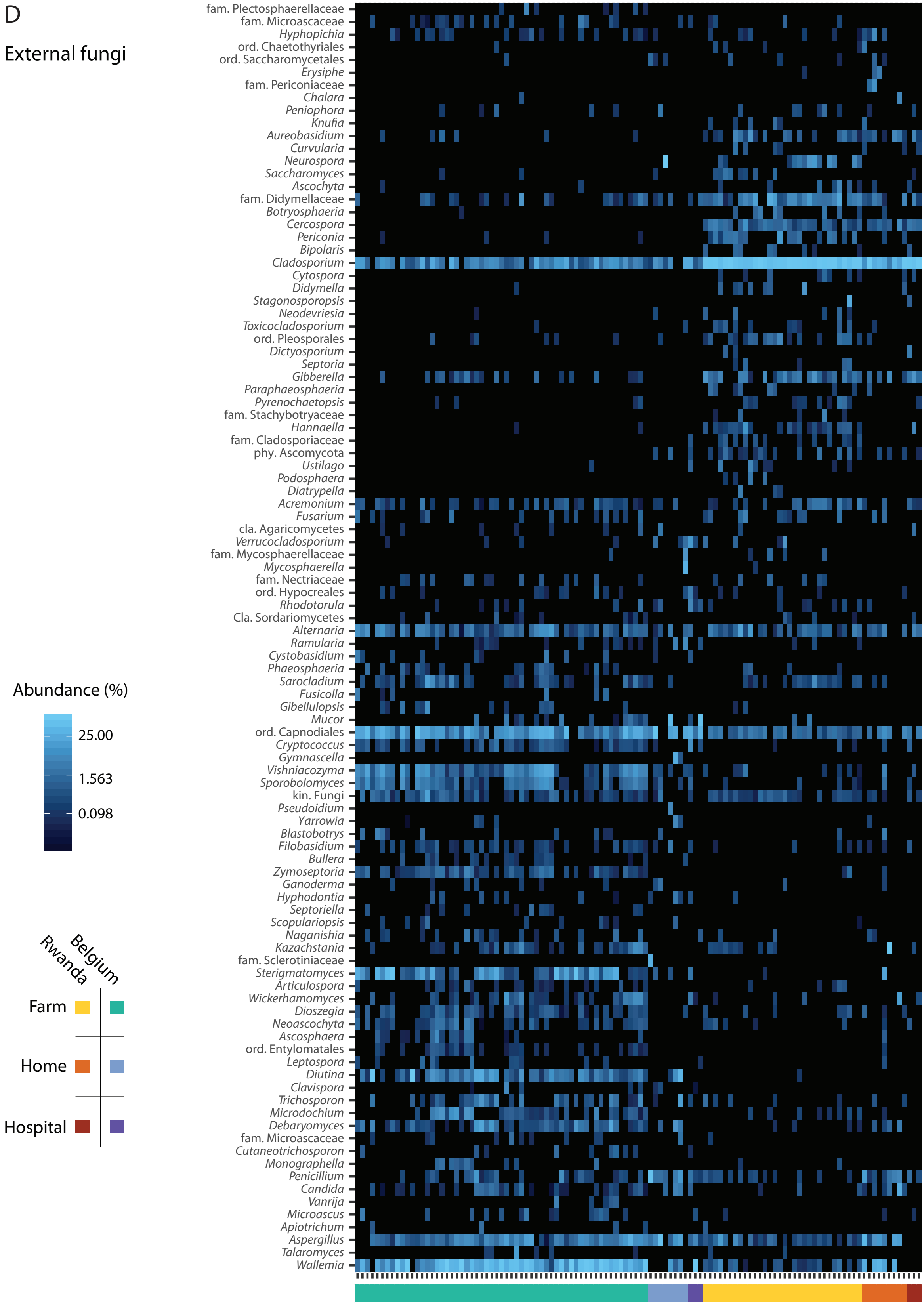
D
External fungi
fam. Plectosphaerellaceae
fam. Microascaceae
Hyphopichia
ord. Chaetothyriales
ord. Saccharomycetales
Erysiphe
fam. Periconiaceae
Chalara
Peniophora
Knufia
Aureobasidium
Curvularia
Neurospora
Saccharomyces
Ascochyta
fam. Didymellaceae
Botryosphaeria
Cercospora
Periconia
Bipolaris
Cladosporium
Cytospora
Didymella
Stagonosporopsis
Neodevriesia
Toxicocladosporium
ord. Pleosporales
Dictyosporium
Septoria
Gibberella
Paraphaeosphaeria
Pyrenochaetopsis
fam. Stachybotryaceae
Hannaella
fam. Cladosporiaceae
phy. Ascomycota
Ustilago
Podosphaera
Diatrypella
Acremonium
Fusarium
cla. Agaricomycetes
Verrucocladosporium
fam. Mycosphaerellaceae
Mycosphaerella
fam. Nectriaceae
ord. Hypocreales
Rhodotorula
Cla. Sordariomycetes
Alternaria
Ramularia
Cystobasidium
Phaeosphaeria
Sarocladium
Fusicolla
Gibellulopsis
Mucor
ord. Capnodiales
Cryptococcus
Gymnascella
Vishniacozyma
Sporobolomyces
kin. Fungi
Pseudoidium
Yarrowia
Blastobotrys
Filobasidium
Bullera
Zymoseptoria
Ganoderma
Hyphodontia
Septoriella
Scopulariopsis
Naganishia
Kazachstania
fam. Sclerotiniaceae
Sterigmatomyces
Articulospora
Wickerhamomyces
Dioszegia
Neoascochyta
Ascosphaera
ord. Entylomatales
Leptospora
Diutina
Clavispora
Trichosporon
Microdochium
Debaryomyces
fam. Microascaceae
Cutaneotrichosporon
Monographella
Penicillium
Candida
Vanrija
Microascus
Apiotrichum
Aspergillus
Talaromyces
Wallemia
Abundance (%)
25.00
1.563
0.098
Rwanda
Belgium
Farm
Home
Hospital

Figure S4

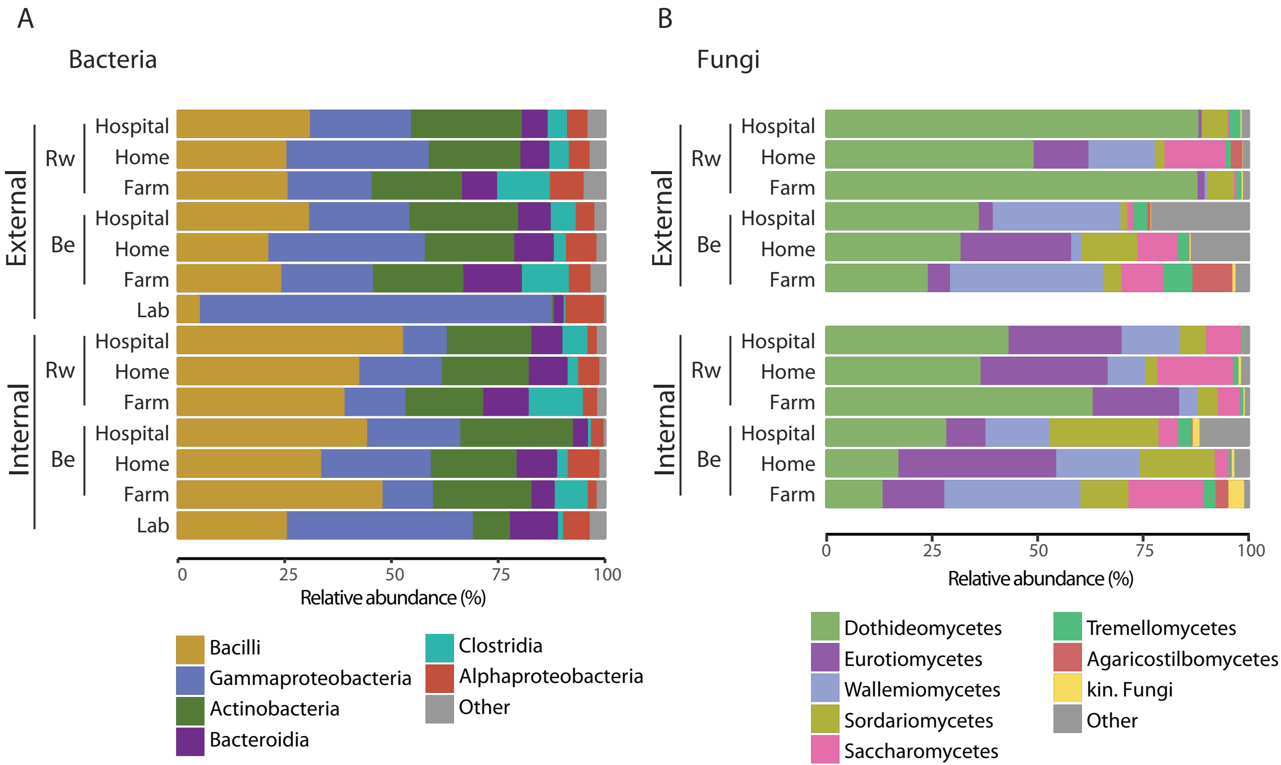
A
Bacteria
External
Internal
Rw
Be
Hospital
Home
Farm
Lab
0
25
50
75
100
Relative abundance (%)
Bacilli
Gammaproteobacteria
Actinobacteria
Bacteroidia
Clostridia
Alphaproteobacteria
Other
B
Fungi
Dothideomycetes
Eurotiomycetes
Wallemiomycetes
Sordariomycetes
Saccharomycetes
Tremellomycetes
Agaricostilbomycetes
kin. Fungi
Other

Figure S5

A

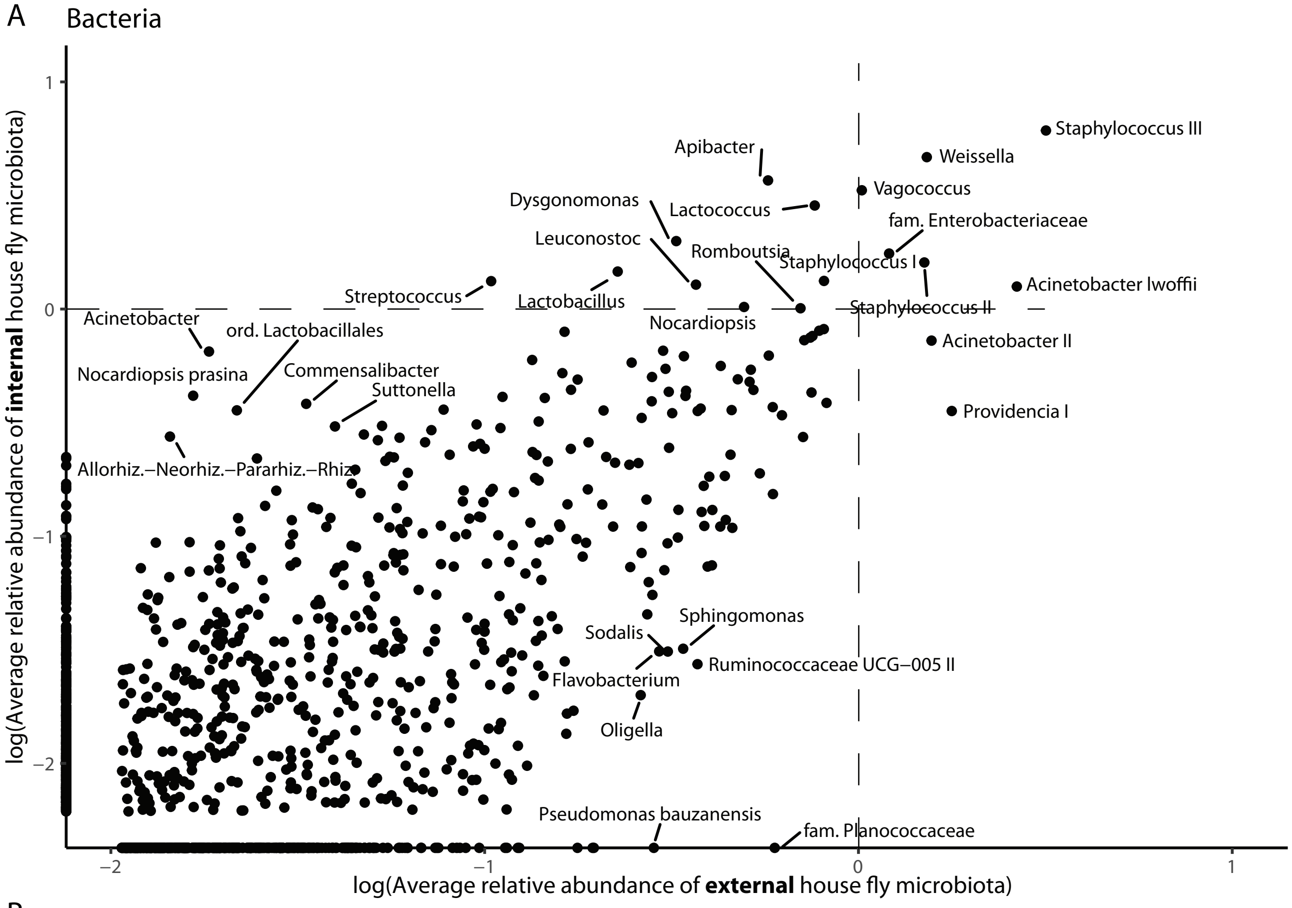

Bacteria
Staphylococcus III
Weissella
Apibacter
Vagococcus
Dysgonomonas
Lactococcus
fam. Enterobacteriaceae
Leuconostoc
Romboutsia
Staphylococcus I
Acinetobacter lwoffii
Streptococcus
Lactobacillus
Staphylococcus II
Acinetobacter
ord. Lactobacillales
Nocardiopsis
Acinetobacter II
Nocardiopsis prasina
Commensalibacter
Suttonella
Providencia I
Allorhiz.−Neorhiz.−Pararhiz.−Rhiz
Sphingomonas
Sodalis
Ruminococcaceae UCG−005 II
Flavobacterium
Oligella
Pseudomonas bauzanensis
fam. Planococcaceae
1
0
−1
−2
−2
−1
0
1
log(Average relative abundance of internal house fly microbiota)
log(Average relative abundance of external house fly microbiota)

B

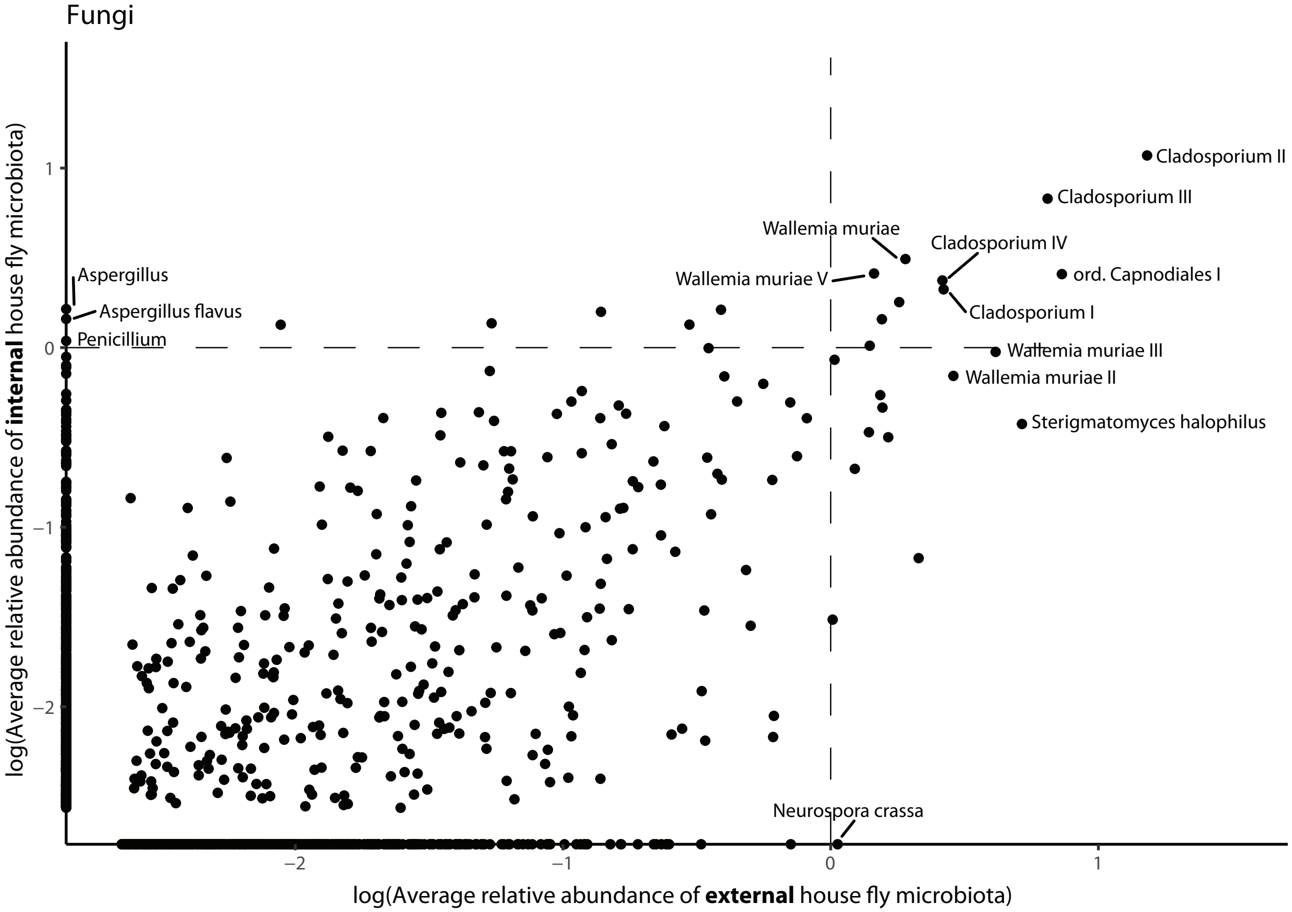

Fungi
Cladosporium II
Cladosporium III
Wallemia muriae
Cladosporium IV
Aspergillus
Wallemia muriae V
ord. Capnodiales I
Aspergillus flavus
Cladosporium I
Penicillium
Wallemia muriae III
Wallemia muriae II
Sterigmatomyces halophilus
Neurospora crassa
1
0
−1
−2
−2
−1
0
1
log(Average relative abundance of internal house fly microbiota)
log(Average relative abundance of external house fly microbiota)

Figure S6

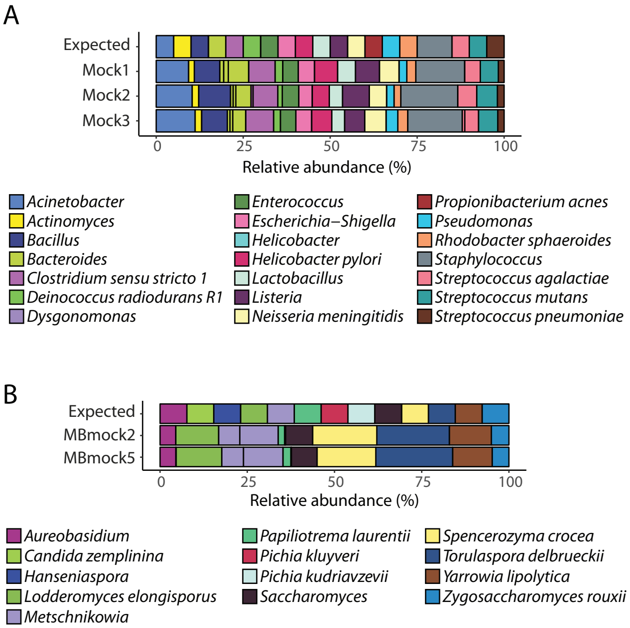
A
Expected
Mock1
Mock2
Mock3
0
25
50
75
100
Relative abundance (%)
Acinetobacter
Actinomyces
Bacillus
Bacteroides
Clostridium sensu stricto 1
Deinococcus radiodurans R1
Dysgonomonas
Enterococcus
Escherichia−Shigella
Helicobacter
Helicobacter pylori
Lactobacillus
Listeria
Neisseria meningitidis
Propionibacterium acnes
Pseudomonas
Rhodobacter sphaeroides
Staphylococcus
Streptococcus agalactiae
Streptococcus mutans
Streptococcus pneumoniae
B
Expected
MBmock2
MBmock5
0
25
50
75
100
Relative abundance (%)
Aureobasidium
Candida zemplinina
Hanseniaspora
Lodderomyces elongisporus
Metschnikowia
Papiliotrema laurentii
Pichia kluyveri
Pichia kudriavzevii
Saccharomyces
Spencerozyma crocea
Torulaspora delbrueckii
Yarrowia lipolytica
Zygosaccharomyces rouxii
