# Supplementary material for: Microbial communities of the house fly Musca domestica vary with geographical location and habitat
Source: Microbiome. 2019 Nov 8;7:147. doi: 10.1186/s40168-019-0748-9 (PMC6839111; doi:10.1186/s40168-019-0748-9)
Supplement: Supplementary file 3 — Additional file 3: Table S2. Alpha diversity measured by Shannon index and observed amplicon sequence variants (ASVs) of the grouped samples. Table S3. The prevalence and abundance of pathogenic bacteria from house flies displaying location specific patterns. Initial analysis and selection was based on Table 1 in Khamesipour et al 2018 [16] enlisting main pathogenic bacterial genera and species observed in house flies. Table S6. Composition of the mock communities. [file 40168_2019_748_MOESM3_ESM.docx]

**Supplemental Table S2. Alpha diversity measured by Shannon index and observed amplicon sequence variants (ASVs) of the grouped samples**

| Shannon |  |  |  |  | Kruskal Wallis | | |
| --- | --- | --- | --- | --- | --- | --- | --- |
|  | Group 1 | Median shannon | Group 2 | Median shannon | H | p-value | q-value |
| Sex | Female | 5.0 | Male | 5.1 | 0.826514 | 0.36328 | 0.4541 |
| Compartment | External | 6.2 | Internal | 4.3 | 191.2987 | 1.66E-43 | 1.66E-42 |
| Country | Belgium | 5.2 | Rwanda | 5.0 | 6.121361 | 0.01336 | 0.03339 |
| Environment | Farm | 5.5 | Home | 4.9 | 19.0266 | 1.29E-05 | 7.2E-05 |
|  | Farm | 5.5 | Hospital | 4.4 | 25.36913 | 4.73E-07 | 7E-06 |
|  | Home | 4.9 | Hospital | 4.4 | 2.086678 | 1.49E-01 | 0.18574 |

| Observed ASVs |  |  |  |  | Kruskal Wallis | | |
| --- | --- | --- | --- | --- | --- | --- | --- |
|  | Group 1 | Median observed | Group 2 | Median observed | H | p-value | q-value |
| Sex | Female | 127.5 | Male | 135.0 | 0.580327 | 0.44618 | 0.47805 |
| Compartment | External | 191.0 | Internal | 107.5 | 119.4839 | 8.21E-28 | 8.21E-27 |
| Country | Belgium | 136.0 | Rwanda | 129.5 | 1.114163 | 0.29118 | 0.31814 |
| Environment | Farm | 158.5 | Home | 125.0 | 14.37144 | 1.50E-04 | 0.00032 |
|  | Farm | 158.5 | Hospital | 115.5 | 17.76227 | 2.50E-05 | 9.4E-05 |
|  | Home | 125.0 | Hospital | 115.5 | 0.727347 | 3.94E-01 | 0.39374 |

**Supplemental Table S3.** The prevalence and abundance of pathogenic bacteria from house flies displaying location specific patterns. Initial analysis and selection was based on Table 1 in Khamesipour et al. [16] enlisting main pathogenic bacterial genera and species observed in house flies.

|  | Prevalence (%) | | | Abundance (%) | | |
| --- | --- | --- | --- | --- | --- | --- |
|  | Farms | Homes | Hospitals | Farms | Homes | Hospitals |
| gen. *Streptococcus* | 51.3 | 75.0 | 92.5 | 2.23 | 0.50 | 6.81 |
| gen. *Micrococcus* | 17.4 | 36.6 | 58.1 | 0.19 | 0.06 | 0.63 |
| gen. *Clostridium* | 88.9 | 52.7 | 58.8 | 0.49 | 1.62 | 0.86 |
| gen. *Escherichia-Shigella* | 50.0 | 21.0 | 43.8 | 0.10 | 0.88 | 0.53 |
| *Streptococcus pyogenes* | 0.0 | 0.3 | 16.3 | 0.00 | 0.00 | 2.20 |

**Supplemental Table S6. Composition of the mock communities**

|  | Bacterial mock community | Fungal mock community |
| --- | --- | --- |
| 1 | *Acinetobacter baumannii* | *Aureobasidium pullulans* |
| 2 | *Actinomyces odontolyticus* | *Candida zemplinina* |
| 3 | *Bacillus cereus* | *Hanseniaspora opuntiae* |
| 4 | *Bacteroides vulgatus* | *Lodderomyces elongisporus* |
| 5 | *Clostridium beijerinckii* | *Metschnikowia pulcherrima* |
| 6 | *Deinococcus radiodurans* | *Papiliotrema laurentii* |
| 7 | *Enterococcus faecalis* | *Pichia kluyveri* |
| 8 | *Escherichia coli* | *Pichia kudriavzevii* |
| 9 | *Helicobacter pylori* | *Saccharomyces cerevisiae* |
| 10 | *Lactobacillus gasseri* | *Spencerozyma crocea* |
| 11 | *Listeria monocytogenes* | *Torulaspora delbrueckii* |
| 12 | *Neisseria meningitidis* | *Yarrowia lipolytica* |
| 13 | *Propionibacterium acnes* | *Zygosaccharomyces rouxii* |
| 14 | *Pseudomonas aeruginosa* |  |
| 15 | *Rhodobacter sphaeroides* |  |
| 16 | *Staphylococcus aureus* |  |
| 17 | *Staphylococcus epidermidis* |  |
| 18 | *Streptococcus agalactiae* |  |
| 19 | *Streptococcus mutans* |  |
| 20 | *Streptococcus pneumoniae* |  |
